# Multitrophic Interaction in the Rhizosphere of Maize: Root Feeding of Western Corn Rootworm Larvae Alters the Microbial Community Composition

**DOI:** 10.1371/journal.pone.0037288

**Published:** 2012-05-22

**Authors:** Flavia Dematheis, Ute Zimmerling, Cecilia Flocco, Benedikt Kurtz, Stefan Vidal, Siegfried Kropf, Kornelia Smalla

**Affiliations:** 1 Julius Kühn-Institut - Federal Research Centre for Cultivated Plants (JKI), Institute for Epidemiology and Pathogen Diagnostics, Braunschweig, Germany; 2 Department of Crop Science, Agricultural Entomology, Georg-August-University Göttingen, Göttingen, Germany; 3 Department of Biometry and Medical Informatics, Otto-von-Guericke-Universität Magdeburg, Magdeburg, Germany; Argonne National Laboratory, United States of America

## Abstract

**Background:**

Larvae of the Western Corn Rootworm (WCR) feeding on maize roots cause heavy economical losses in the US and in Europe. New or adapted pest management strategies urgently require a better understanding of the multitrophic interaction in the rhizosphere. This study aimed to investigate the effect of WCR root feeding on the microbial communities colonizing the maize rhizosphere.

**Methodology/Principal Findings:**

In a greenhouse experiment, maize lines KWS13, KWS14, KWS15 and MON88017 were grown in three different soil types in presence and in absence of WCR larvae. Bacterial and fungal community structures were analyzed by denaturing gradient gel electrophoresis (DGGE) of the16S rRNA gene and ITS fragments, PCR amplified from the total rhizosphere community DNA. DGGE bands with increased intensity were excised from the gel, cloned and sequenced in order to identify specific bacteria responding to WCR larval feeding. DGGE fingerprints showed that the soil type and the maize line influenced the fungal and bacterial communities inhabiting the maize rhizosphere. WCR larval feeding affected the rhiyosphere microbial populations in a soil type and maize line dependent manner. DGGE band sequencing revealed an increased abundance of *Acinetobacter calcoaceticus* in the rhizosphere of several maize lines in all soil types upon WCR larval feeding.

**Conclusion/Significance:**

The effects of both rhizosphere and WCR larval feeding seemed to be stronger on bacterial communities than on fungi. Bacterial and fungal community shifts in response to larval feeding were most likely due to changes of root exudation patterns. The increased abundance of *A. calcoaceticus* suggested that phenolic compounds were released upon WCR wounding.

## Introduction

*Diabrotica virgifera virgifera* (Western Corn Rootworm, WCR; *Coleoptera: Chrysomelidae*), is considered one of the most destructive agricultural pests of maize (*Zea mays* L.) in the US [1]. Since the beginning of the 1980s the WCR was accidentally and repeatedly introduced into Europe [2], [3], infesting more than 20 countries by the end of 2010 [4]. In case of establishment of this pest in European maize growing regions, damage costs are expected of about 450 million Euro per year [5]. Major damages are caused by the larvae feeding on the maize roots [6], resulting in disrupted water and nutrient uptake [7] and, at high larval densities, in plant lodging [8]. Due to the expected severe yield losses the EU established mandatory eradication and containment measures since 2003 [9], [10]. Following overwintering, the larvae hatch in the soil and start to feed on roots, preferably of maize plants. They pass through three larval stages before pupating in the soil. In July adult beetles begin to emerge from the soil and to feed on corn foliage, silks, pollen, and ear tips. Ovideposition starts in July-August, and traditionally the females lay the eggs in the soil near the base of maize plants [11]. Crop rotation was considered for almost a century the most effective and environmentally benign WCR management option [12]. Nowadays, several American WCR populations have lost ovipositional fidelity to maize fields [13] bypassing the corn-soybean crop rotation strategy. Additionally, this pest species showed resistances against insecticides [14] and Bt-transgenic plants expressing CryBb1 [15], [16]. Therefore, a better understanding of the ecology of this soil-dwelling pest and its multitrophic interactions in the rhizosphere of their maize host plants [6] is needed in order to develop refined pest control strategies.

Beneficial rhizosphere microorganisms promote plant growth and health by nutrient solubilization, nitrogen fixation, and plant hormone production [17]. In addition, these microorganisms are involved in plant disease suppression or in the reduction of herbivorous insect damage [18], [19], either through a direct antagonism of soil-borne pathogens or triggering plant-mediated resistance responses [18], [20].

Several studies have shown that the microbial composition in the rhizosphere may be influenced by different biotic and abiotic factors such as soil type, climate, cropping history, plant species, plant developmental stage, and to a lesser extent cultivar [21]. Furthermore, it has been shown that root-feeding pests such as leather jacket larvae (*Tipula paludosa*) or cyst nematodes (*Heterodera trifolii*) resulted in shifts in the microbial communities colonizing the rhizosphere, most likely by changes of root exudation patterns [22], [23], [24], [25]. Root exudates, being suitable substrates for a wide range of microorganisms, were shown to play a fundamental role in shaping the microbial populations in the plant rhizosphere [26], [27], [28].

Despite the importance of the rhizosphere microorganisms, little is known about the multitrophic interactions between plants, microbial communities in the rhizosphere and root feeding insects. To our knowledge, only Prischmann et al. [29] provided information on the interaction of WCR and the maize rhizosphere bacterium *Serratia* by means of a cultivation-dependent method.

In this study we aimed at unravelling the effects of WCR root feeding on fungal and bacterial communities in the maize rhizosphere. Because different soil types and different maize lines might support different rhizosphere microbial communities, a greenhouse experiment was performed using three different soil types (Haplic Chernozem, Haplic Luvisol and Eutric Vertisol) and four maize lines including KWS13, KWS14 and KWS15, and the transgenic maize MON88017. Different maize lines were also chosen because Broekgaarden et al. [30] observed that the same plant species, in response to the same herbivorous insect, may activate cultivar-dependent transcriptomic changes, which might affect the rhizosphere microbial communities. The transgenic maize expressing the insecticidal Cry3Bb1 protein from *Bacillus thuringiensis* subsp. *kumamotoensis* was included in our study, as detrimental effects on larval survival were expected. Moreover, feeding behaviour of WCR larvae considerably differs between conventional and transgenic cultivars [31]. We hypothesize that in response to WCR feeding, changes in the root exudation will result in shifts of the microbial communities in the rhizosphere of maize according to the soil type and maize line combination, and these effects would be less pronounced in the rhizosphere of the transgenic maize MON88017. The effects of larval feeding on dominant bacterial and fungal populations in the rhizosphere were analyzed by denaturing gradient gel electrophoresis (DGGE) of 16S rRNA gene (bacteria) and ITS fragments (fungi) amplified from total community (TC) DNA.

## Materials and Methods

### Experimental design

A greenhouse experiment was performed under quarantine conditions as in Germany WCR is listed as a quarantine organism. Seeds of each maize line were sown in plastic trays (34 cm×26 cm) containing three different soil types (Haplic Chernozem, Haplic Luvisol and Eutric Vertisol) and the seedlings were transferred one week later into pots (Ø 13 cm). A gauze (voile, 100% polyester, Alfatex Göttingen, Germany) was glued to the bottom of the pots to avoid the escape of larvae. For each plant line four independent replicates per soil type were prepared. After three weeks of growing (plant developmental stage V3), circa 60 eggs of WCR were injected close to the stem at 5 cm depth. After 20 days of larval feeding the plants were harvested and the rhizosphere isolated from the maize roots for total community DNA extraction and molecular analysis.

### Soil types

The three agricultural soil types Haplic Chernozem, Haplic Luvisol and Eutric Vertisol were collected nearby Göttingen (Germany) in June 2008 (Table S1). 400 kg of each soil type were taken from four different spots per field, five meters apart from each other, along a transect. The soils were taken to a depth of 25 cm. In order to avoid any alteration of the microbial content, the soils were immediately transported to the laboratory, homogenized by a soil crusher machine (Unifix 300, Möschle, Ortenberg, Germany), and sieved through a 10 mm mesh to remove stones and plant residues. The majority of the soil was used for the greenhouse experiment, while little volumes were collected in four falcon tubes (50 mL) per soil type and used as replicates to investigate the soil microbial composition.

### Maize lines and growth conditions

The maize lines used in this study were three Northern European maize breeding lines provided by the seed company KWS (Einbeck, Germany: KWS13, KWS14, KWS15) and the transgenic maize MON88017 (Monsanto, St. Louis, MO, USA). The KWS flint x dent varieties differ in their maturity classes (with KWS 13 being the earliest and KWS15 the latest developing maize line), and thus also in their root exudation patterns. The genetically modified maize was developed to express two proteins: the insecticidal Cry3Bb1 protein from *Bacillus thuringiensis* subsp. *kumamotoensis*, and the CP4 EPSPS protein from *Agrobacterium* sp. conferring glyphosate tolerance. According to the Canadian Food Inspection Service, the Cry3Bb1 protein is locally expressed in root tissues with concentrations of 100–370 µg g^−1^ dry weight root tissue [32].

The maize growing conditions adopted in the greenhouse were as follows: 40% relative humidity, 24°C mean temperature and 16 h of additional illumination with sodium lamps (400W, HS2000, Hortilux Schréder, Monster, The Netherlands). The pots of plants grown in the same soil were placed in the same tray that was moved twice a week in the greenhouse to randomize the growing conditions. The fertilizer Hakaphos blau (Compo, Münster, Germany; 2.5%) was applied to plants older than 14 days by watering once a week.

### WCR egg inoculum

WCR eggs of the non-diapausing strain were provided by USDA-ARS (Northern Grain Insect Research Laboratory, Brookings, SD, USA) and stored at 8°C until their use. In order to stimulate the larval development, the eggs were incubated at 26°C, 60% relative humidity in dark conditions for 12 days and checked for visible larvae presence using a dissecting microscope. Afterwards the eggs were washed in a sieve (Ø 250 µm) and suspended in 0.15% agar solution. Hatch tests were prepared to assess the hatch times and the hatch rates as follows: 0.5 mL of egg suspension were applied on a sterile humid filter paper and incubated at the same conditions as described for larval development. The eggs were counted and checked daily for hatching. The mean values estimated for the hatch time and hatch rate were two days and 72%, respectively.

### Rhizosphere sampling and microbial cells extraction

Six-week old maize plants were removed from the soil and shaken vigorously. The soil tightly adhering to the roots was considered as rhizosphere and collected using a Stomacher blender (Stomacher 400, Seward, England) as described by Costa et al. [33]. The microbial pellets were harvested by centrifugation at 10,000 *g* at 4°C for 30 min and homogenized with a spatula.

### Total community DNA extraction

The TC DNA was extracted from 0.5 g of soil and from 0.5 g of a rhizosphere pellet. The cells were lysed mechanically twice with the FastPrep FP120 bead beating system (Q-Biogene, Carlsbad, CA, USA) for 30 s at high speed. Thereafter the DNA was extracted with the FastDNA SPIN Kit for Soil (Q-Biogene, Carlsbad, CA, USA) according to the instructions of the manufacturer. The extracted DNA was purified with the GENECLEAN SPIN Kit (Q-Biogene, Heidelberg, Germany) according to the manufacturer's protocol. The TC DNA was checked on 0.8% agarose gel and DNA concentrations were estimated visually using the quantitative marker High DNA Mass Ladder (Invitrogen). Genomic DNA samples were differently diluted in MilliQ sterilized water to obtain ca. 20 ng/µL DNA for use as a PCR template.

### PCR amplification of the Internal Transcribed Spacer (ITS) regions and 16S rRNA gene fragments

The ITS fragments of the fungal communities were directly amplified from TC DNA extracted from soil and rhizosphere samples obtained from plants grown with or without WCR larvae. The ITS amplification was performed using a nested PCR approach with the primer pair ITS1F/ITS4 and ITS2/ITS1F-GC according to Weinert et al. [34]. The same TC DNA samples extracted from soil and plant rhizosphere were used to amplify the 16S rRNA gene fragments using the primer pair F984GC/R1378 [35]. Reaction mixture and PCR conditions applied were described by Costa et al. [33].

### Denaturing Gradient Gel Electrophoresis (DGGE)

The DGGE analyses of the fungal and bacterial communities were carried out in the PhorU2 machine (Ingeny, Goes, The Netherlands). The DGGE gels were prepared as described by Weinert et al. [34]. Gels were silver stained and air dried according to Heuer et al. [36]. Gel images were digitally captured using an Epson 1680 Pro scanner (Seiko-Epson, Japan) with high resolution setting.

### DGGE data analysis and statistical testing

DGGE profiles which represent the fingerprint of dominant bacterial or fungal populations were analyzed with the software package GELCOMPAR II 4.5 (Applied Math, Ghent, Belgium) as described by Gomes et al. [37]. Cluster analyses (UPGMA) based on the Pearson correlation indices (which consider band presence/absence and relative abundance) were performed to evaluate the percentage of similarity shared among samples. Pair-wise statistical analyses (Permutation tests) were applied on the values of the similarity matrix according to Kropf et al. [38]. The differences between groups (*D* values) and significant values (*P* values<0.05) are always reported. Furthermore, an extension of the permutation method described in Kropf et al. [38] for two-factorial designs was developed and used to analyze interaction among larvae, maize line and bacterial or fungal communities.

### Identification of the bacterial populations behind differentiating DGGE bands

In order to identify the main bacterial population responding to WCR feeding, bands with increased intensity in the treatments with WCR larvae were excised, re-amplified and sequenced. Pieces of the central part of the band excised from the acrylamide gel were transferred in 1.5 mL tubes, combining replicates of band 1 per maize line and soil type. Gel slices were crushed with the top of a sterile tip and the contained DNA was suspended into sterile TE buffer, pH 8, by overnight incubation at 4°C. After centrifugation at 11,000× *g* for 60 s, the supernatant containing the band DNA was transferred to a new tube and 1 µL of it was used as template for a new PCR reaction. The PCR was performed using the same conditions described for the bacterial community amplification, except for the use of a forward primer without GC-clamp (F984). PCR products were ligated in the pGEM-T vector system (Promega) and transformed into *Escherichia coli* (JM109 Competent Cells, Promega) according to the manufacturer's instructions. The clones were re-amplified with the primer pair T7/SP6 to select the transformants carrying the insert with the expected size. The T7/SP6 amplicons of the positive clones were re-amplified with the primers F984-GC/R1378 to identify on DGGE gels the clones carrying an insert with identical electrophoretic mobility of the excised band. For each maize line and soil type combination three to four clones per DGGE band were sequenced. 16S rRNA gene sequences were analyzed using BLAST-n program at the NCBI site.

#### Nucleotide sequence accession numbers

Nucleotide sequences determined in this study were deposited in the GenBank database under accession numbers JN836602 to JN836633.

## Results

### Soil type and maize plant line shape the bacterial and fungal community composition in the rhizosphere

**Figure 1:**
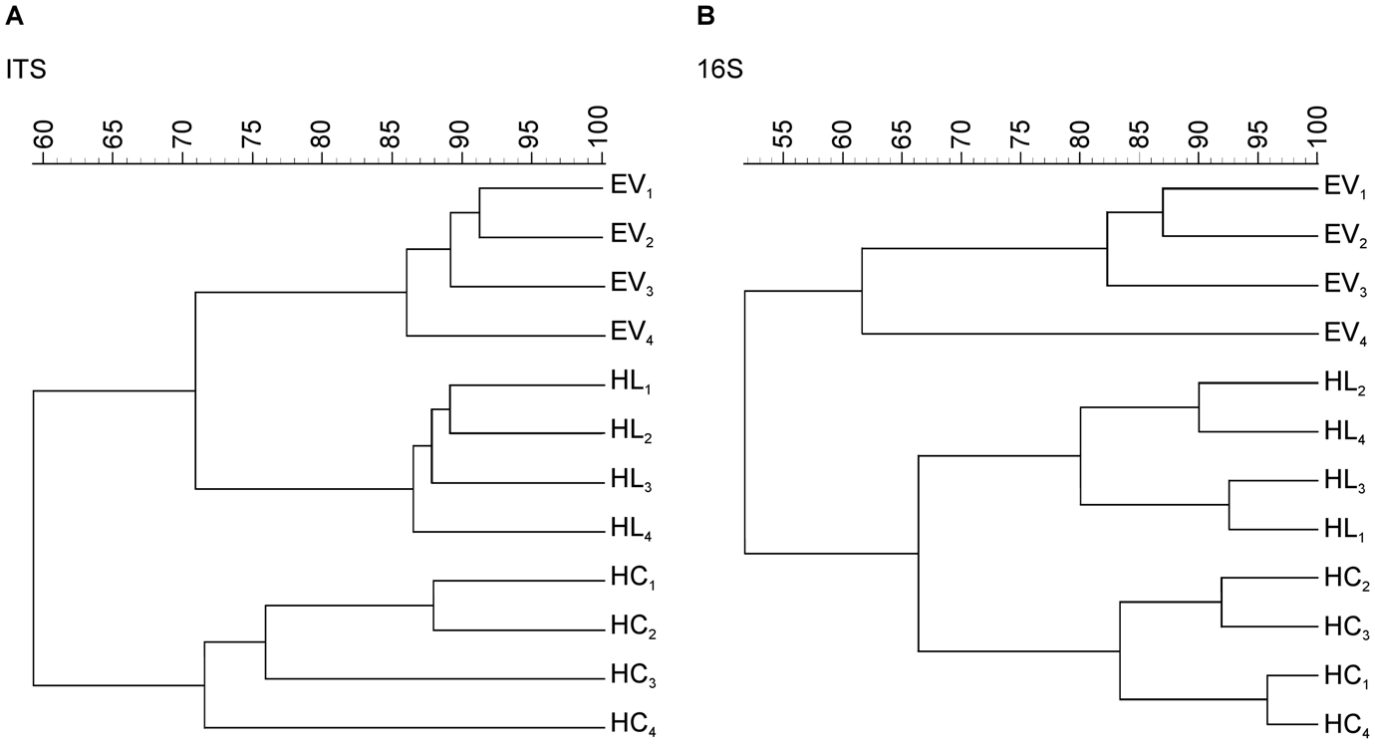
UPGMA dendrograms of DGGE fingerprints of 16S rRNA gene and ITS fragments PCR-amplified from TC DNA of three different soil types. HC: Haplic Chernozem; HL: Haplic Luvisol; EV: Eutric Vertisol. A: UPGMA of fungal fingerprints; B: UPGMA of bacterial fingerprints. Independent replicates are labeled 1 to 4. The dendrograms were constructed using the Pearson correlation coefficient. The scale shows similarity values.

In order to verify the hypothesis that Haplic Chernozem, Haplic Luvisol and Eutric Vertisol support different microbial communities, fungal and bacterial populations in these soils were investigated and compared by means of DGGE fingerprints. Both fungal and bacterial DGGE fingerprints revealed complex patterns with ca. 40 bands for each soil type and differences in the relative abundance of several microbial populations among soils (data not shown). UPGMA dendrograms of fungal and bacterial communities showed that the different soil types distinctly clustered apart (Fig. 1A and B) and permutation testing confirmed that the soil type dependent differences were significant (*P*<0.04). The high dissimilarity (*D*>16) of both fungal and bacterial populations inhabiting the three soils suggested a soil type specific microbial community structure (Table S2).

**Figure 2:**
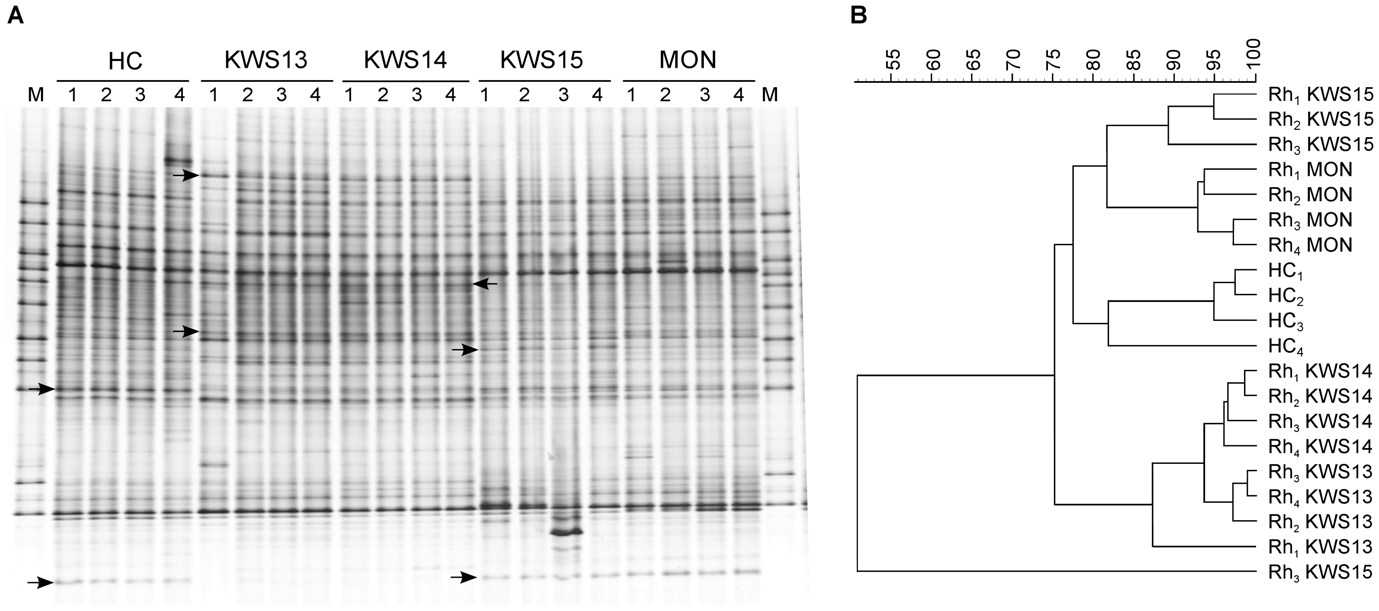
DGGE fingerprints of ITS fragments PCR-amplified from TC DNA extracted from soil and rhizosphere samples and corresponding UPGMA dendrogram. (A) DGGE fingerprints of dominant fungal populations in Haplic Chernozem (HC) soil and in the maize rhizosphere of KWS13, KWS14, KWS15 and MON88017 grown in the same soil type. The independent replicates are labeled 1 to 4. M: fungal marker prepared with the ITS fragments amplified from *Verticillium nigrescens*, *Basidiomycete* sp., *Trichoderma* sp., *Doratomyces* sp., *Verticillium dahliae*, *Penicillium canescens*, *Fusarium graminearum*, *Nectria haematococca*, *Fusarium solani*, *Fusarium redolens*, and *Sclerotinia sclerotiorum*. Arrows indicate maize genotype effects. (B) UPGMA dendrogram constructed using the Pearson correlation coefficient. The scale shows similarity values. Rh: rhizosphere samples.

To study the influence of the four maize lines used in our experiment (KWS13, KWS14, KWS15, and MON88017) on the soil microbial composition, fungal and bacterial fingerprints of bulk soil and corresponding rhizosphere samples of the four maize lines were compared. The DGGE patterns of fungal communities in the bulk soil and in the rhizosphere of KWS13, KWS14, KWS15, and MON88017 are exemplarily shown for Haplic Chernozem in Fig. 2A. The cluster analysis of all DGGE gels revealed that the fungal communities of bulk soil samples clustered always separately from the rhizosphere samples (e.g. Fig. 2B). The fungal composition of soil and rhizosphere patterns of each maize line in all three soil types were statistically different (*P* = 0.03), with *D* values ranging between 3 and 17.2 (Table S3). Similar to the fungal communities, the comparisons of the bacterial fingerprints between bulk soil and rhizosphere samples revealed for all the maize lines significant rhizosphere effects (*P* = 0.03) in all three soil types investigated, with *D* values ranging between 10 and 58. In contrast to the fungal communities, a higher dissimilarity in the bacterial community composition between bulk soil and rhizosphere samples was observed (Table S3).

In order to test the hypothesis that different maize lines differently affect the rhizosphere microbial communities, pair-wise comparisons of the rhizosphere DGGE fingerprints of KWS13, KWS14, KWS15 and MON88017 grown in the same soil type were performed, and differences were tested for significance. The pair-wise comparison of the rhizosphere fungal fingerprints obtained from different maize lines showed line-dependent differences in the relative abundance of several fungal populations in all three soils (e.g. Fig. 2A). Although a clustering between the plant lines was not always observed, likely due to the variability among replicates, UPGMA-dendrograms of the fungal communities revealed, independently from the soil type, always two groups KWS13/KWS14 and KWS15/MON88017 (e.g. Fig. 2B). The statistical analysis showed significant differences (*P* = 0.03) of the rhizosphere fungal populations between all maize lines in all three soil types, except between KWS13 and KWS14 in Haplic Chernozem, and between KWS15 and MON88017 in Haplic Luvisol (Table 1). Small differences of the rhizosphere fungal communities between KWS13 and KWS14 (2<*D* values<6.2), and between KWS15 and MON88017 (1.2<*D* values<9.3) were observed in all three soil types. Pair-wise comparison of rhizosphere bacterial fingerprints obtained from different maize lines revealed different bacterial community structures among maize lines in all soil types (data not shown). UPGMA-cluster analysis showed that the bacterial communities in the rhizosphere of each maize line clustered apart from each other in all soil types, except for KWS13 and KWS14 in Haplic Chernozem, and for KWS14 and KWS15 in Haplic Luvisol, which formed a mixed cluster due to high variability within KWS13 and KWS14 replicates (data not shown). Bacterial community patterns obtained from the rhizosphere of MON88017 clustered separately from those of the other maize lines in both Haplic Chernozem and Luvisol. In Eutric Vertisol, KWS15 and MON88017 formed one cluster sharing low similarity (36%). Statistical testing revealed significant differences (*P* = 0.03) between the bacterial communities in the rhizosphere between all maize lines, except for KWS14/KWS15 in Haplic Luvisol (Table 1). Thus, the bacterial communities in the maize rhizosphere, as well as the fungal communities, were influenced by the maize line in a soil type specific manner.

**Table 1:** Percentage dissimilarity (*D*) and significant values (*P*) of rhizosphere fungal or bacterial fingerprints between different maize lines (KWS13, KWS14, KWS15 and MON88017) grown in the soil types Haplic Chernozem, Haplic Luvisol, and Eutric Vertisol.

	Haplic Chernozem		Haplic Luvisol		Eutric Vertisol	
	*D*	*P*	*D*	*P*	*D*	*P*
***Fungi***						
KWS13/KWS14	2.2	0.06	6.2	**0.03**	2	**0.03**
KWS13/KWS15	14	**0.03**	14.1	**0.03**	8.7	**0.03**
KWS13/MON	21.2	**0.03**	16.6	**0.03**	8.8	**0.03**
KWS14/KWS15	14.8	**0.03**	18.7	**0.03**	11.1	**0.03**
KWS14/MON88017	21.7	**0.03**	17.8	**0.02**	8.6	**0.03**
KWS15/MON88017	9.3	**0.03**	1.2	0.3	5.5	**0.03**
***Bacteria***						
KWS13/KWS14	9.3	**0.03**	15.4	**0,03**	30	**0.03**
KWS13/KWS15	18.1	**0.03**	27.3	**0.03**	50	**0.03**
KWS13/MON88017	15	**0.03**	26.6	**0.03**	65.2	**0.03**
KWS14/KWS15	17.5	**0.03**	9.5	0.06	50.6	**0.03**
KWS14/MON88017	14.2	**0.03**	24.3	**0.03**	56.8	**0.03**
KWS15/MON88017	27.2	**0.03**	16.9	**0.03**	12.8	**0.03**

Values of *P*<0.05 indicate significant differences between rhizosphere samples of different maize lines grown in the same soil type. Permutation testing was done with 10.000 simulations. Bold values indicate significant differences.

### WCR larval feeding effects on the fungal communities in the rhizosphere of maize

**Figure 3:**
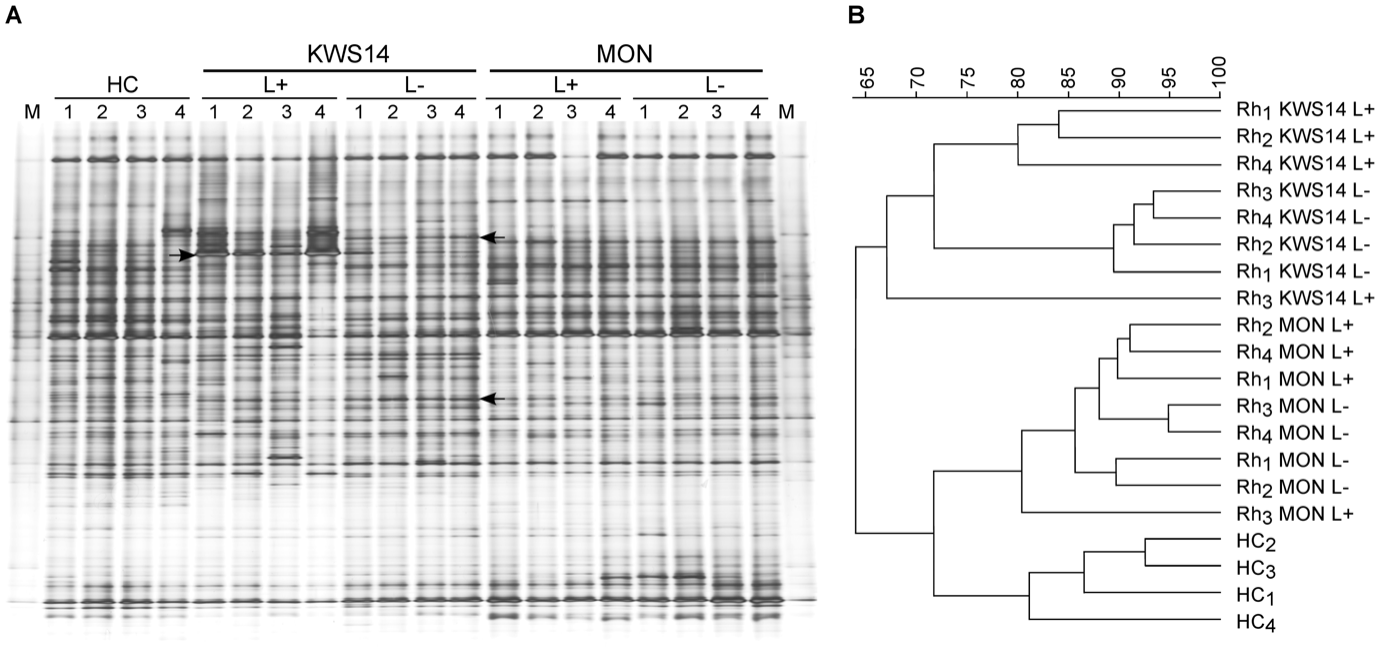
DGGE fingerprints of ITS fragments PCR-amplified from TC DNA extracted from soil and rhizosphere samples, and corresponding UPGMA dendrogram. (A) DGGE profiles of dominant fungal populations in Haplic Chernozem (HC) soil and in the maize rhizosphere of KWS14 and MON88017 grown in the same soil type in presence (L+) or absence (L−) of WCR larvae. Independent replicates are labeled 1 to 4. M: Fungal marker. Arrows indicate WCR larval effects on the rhizosphere fungal communities. (B) UPGMA dendrogram generated by cluster analysis of Pearson's similarity indices. The scale shows similarity values.

The effects of WCR larval feeding on the rhizosphere fungal communities was investigated for all maize lines grown in three soil types by comparing the DGGE fingerprints of the treatments with or without larvae. Only in the fungal fingerprints of KWS14 grown in Haplic Chernozem a pronounced shift upon larval feeding was observed (see arrow in Fig. 3A). In the same soil type minor variations of the fungal communities due to larval presence and activity were observed in the rhizosphere of KWS13, while no shifts were visible in the rhizosphere of KWS15 and MON88017 between samples with (L+) and without (L−) larvae (data not shown). UPGMA dendrograms showed that the fungal communities in the rhizosphere of KWS14 of the (L+) and (L−) treatments grouped separately (Fig. 3B). Although the patterns of KWS13 (L+) and (L−) shared a high similarity (82.4%), separate clusters for treatments with and without larvae were still found (data not shown). In contrast, the fungal communities in the rhizosphere of KWS15 (L+) and (L−) grouped together as well as the rhizosphere fungal populations of MON88017 (L+) and (L−). Permutation testing revealed significant differences of the fungal communities between treatments with or without larvae only in the rhizosphere of KWS13 and KWS14 (*P* = 0.03), indicating a significant effect of the larval feeding on the relative abundance of fungi inhabiting the rhizosphere of these maize lines. Only in the rhizosphere of KWS14 these shifts were highly pronounced (*D* value = 22.8). No significant effect of the larval feeding was observed on the fungal communities in the rhizosphere of KWS15 and MON88017 (Table 2).

**Table 2:** Percentage dissimilarity (*D*) and significance values (*P*) of rhizosphere fungal or bacterial fingerprints between maize lines in presence and in absence of WCR larval feeding (Larvae+/−), in the soil types Haplic Chernozem, Haplic Luvisol, and Eutric Vertisol.

	Haplic Chernozem		Haplic Luvisol		Eutric Vertisol	
	Larvae+/−		Larvae+/−		Larvae+/−	
	*D*	*P*	*D*	*P*	*D*	*P*
***Fungi***						
KWS 13	5.8	**0.03**	11.1	**0.03**	7	**0.03**
KWS 14	22.8	**0.03**	8.9	**0.03**	3.3	**0.03**
KWS 15	0.9	0.3	3.7	0.06	3.8	0.17
MON88017	2	0.1	2	0.2	5.9	**0.03**
***Bacteria***						
KWS 13	15.8	**0.03**	15.6	**0.03**	15.7	**0.03**
KWS 14	31.3	**0.03**	25.5	**0.03**	48.4	**0.03**
KWS 15	23.6	**0.03**	11.9	**0.03**	25.4	**0.03**
MON88017	6.4	0.06	4.1	**0.03**	19.2	**0.03**

*P* values<0.05 indicate significant differences between rhizosphere samples of the same maize line grown with and without larval feeding in the same soil type. Values obtained by Permutation testing using 10.000 simulations. Values in bold show significant values.

In Haplic Luvisoil and Eutric Vertisol, DGGE profiles displayed little variations in the relative abundance of the fungal populations in the rhizosphere of KWS13 and KWS14 in response to larval feeding. The fungal communities in the rhizosphere of MON88017 showed shifts in response to larval feeding only in Eutric Vertisol. No shifts in presence of larvae were observed in the fingerprinting of the fungal populations in the rhizosphere of KWS15 in both soils. UPGMA dendrograms showed clearly separated clusters of (L+) and (L−) samples in the rhizosphere of KWS13 and KWS14 in both soils and in the rhizosphere of MON88017 in Eutric Vertisol (data not shown). Permutation testing between (L+) and (L−) samples showed highly supported differences (*P* = 0.03) of the fungal communities inhabiting the rhizosphere of KWS13, KWS14 in Haplic Luvisoil and Eutric Vertisol. Unexpectedly, a significant effect of larval feeding on the fungal population was observed in the rhizosphere of MON88017 grown in Eutric Vertisol (Table 2).

### WCR larval feeding effect on the bacterial communities in the rhizosphere of maize

The effects of WCR larval feeding on the bacterial populations in the maize rhizosphere were tested by DGGE analysis of 16S rRNA gene fragments amplified from rhizosphere TC DNA of four different maize lines (KWS13, KWS14, KWS15, MON88017) grown in three soil types (Haplic Chernozem, Haplic Luvisol and Eutric Vertisol) in presence and absence of larval feeding.

**Figure 4:**
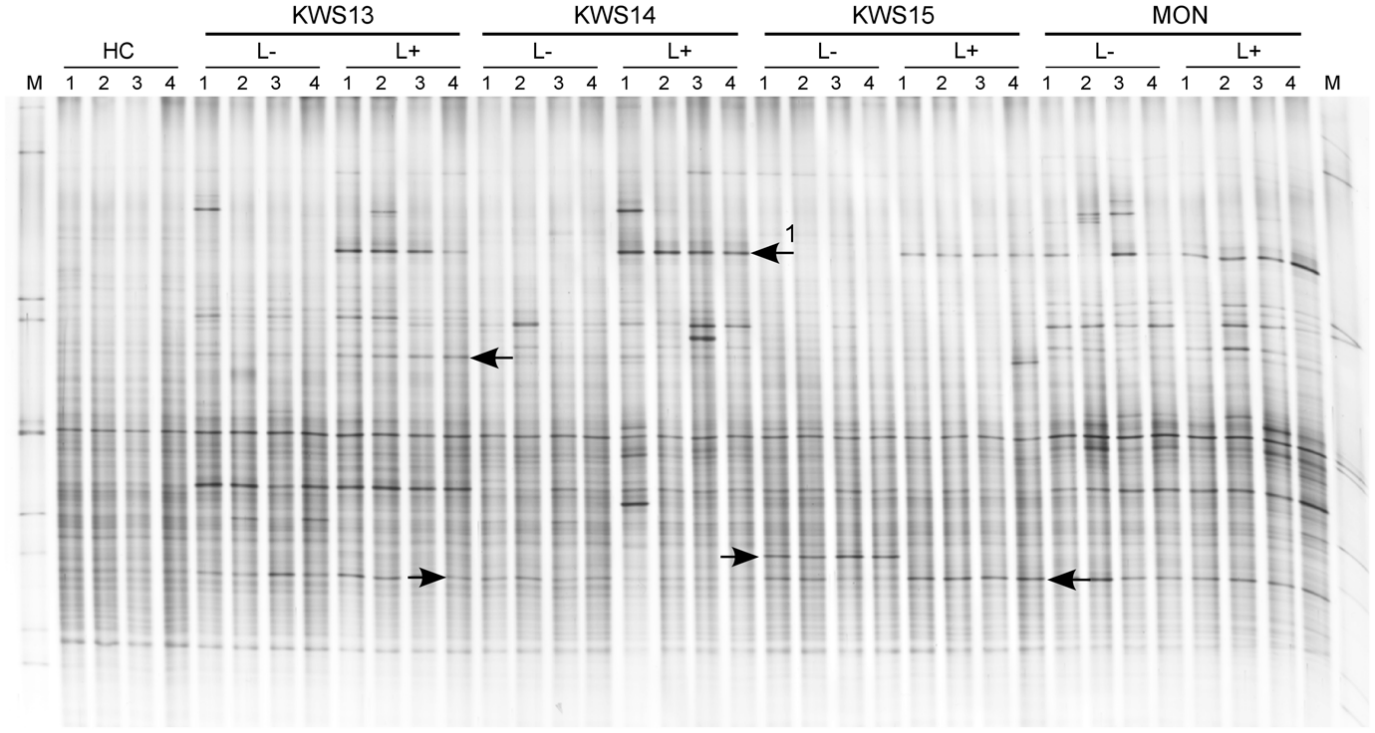
DGGE fingerprints of 16S rRNA gene fragments PCR-amplified from TC DNA extracted from soil and rhizosphere samples, and corresponding UPGMA dendrogram. (A) DGGE profiles of dominant bacterial populations in Haplic Chernozem (HC) soil and in the maize rhizosphere of KWS13, KWS14, KWS15 and MON88017 grown in the same soil type in presence (L+) or absence (L−) of WCR larvae. Independent replicates are labeled 1 to 4. M: Bacterial marker [34]. Arrows indicate WCR larval effects on the rhizosphere bacterial communities.

In Haplic Chernozem pronounced shifts due to WCR larval feeding on the bacterial populations colonizing the maize rhizosphere were observed for all maize lines investigated, except for MON88017 (Fig. 4). The analysis UPGMA showed that the bacterial communities in the rhizosphere of all the KWS lines formed separate clusters (L+) and (L−), although one or two replicates per maize lines did not cluster due to the variability within replicates (data not shown). A mixed cluster was observed for the bacterial rhizosphere populations of MON88017 grown with and without larvae. Permutation testing revealed significant differences of the rhizosphere bacterial communities between (L+) and (L−) samples of KWS13, KWS14, and KWS15 (*P* = 0.03) in Haplic Chernozem, indicating a significant effect of the larval feeding on the bacteria inhabiting the rhizosphere of those maize lines. No effects of the larval feeding were observed on the bacterial communities in the rhizosphere of the transgenic maize MON88017 in Haplic Chernozem.

In Haplic Luvisol and Eutric Vertisol pronounced shifts in the bacterial community patterns were observed upon root larval feeding as well (Table 2). UPGMA dendrograms displayed separate clusters between (L+) and (L−) samples of KWS13, KWS14, KWS15 and MON88017, although one or two replicates did not cluster due to the variability within replicates. Permutation testing revealed significant differences in response to larval feeding in the bacterial populations inhabiting the rhizosphere of all maize lines investigated in Haplic Luvisol and Eutric Vertisol (Table 2).

### Identification of bacteria responding to WCR larval feeding

DGGE fingerprints of the bacterial communities inhabiting the rhizosphere of the four maize lines of (L+) and (L−) treatments in Haplic Chernozem are exemplarily shown in Fig. 4. A dominant band (Band 1, Fig. 4) with identical electrophoretic mobility in the fingerprints of all rhizosphere samples of the (L+) treatments for the KWS lines and of MON88017 (L+) and (L−) treatments was detected. Cloning, sequencing and Blast analysis of this band revealed for most of the clones a high sequence similarity to *Acinetobacter calcoaceticus* (99–100% identity, sequence accession no. JN836603-JN836608, and JN836610-JN836621). Only two clones showed 99% similarity to *Sphingomonas* sp. (accession no. JN836602) and 99% similarity to *Massilia* sp. (accession no. JN836609). A band with the same electrophoretic mobility of Band 1 in Haplic Chernozem was observed in the bacterial fingerprints of KWS13 and KWS14 in Haplic Luvisol (Fig. S1) and of KWS13, KWS15 and MON88017 in Eutric Vertisol (data not shown). Sequencing of this band from the bacterial DGGE fingerprints in the rhizosphere of KWS13 from both soils Haplic Luvisol and Eutric Vertisol revealed again the highest similarity to *Acinetobacter calcoaeticus* (99–100% identity, sequence accession no. JN836622-JN836629). Bacterial community fingerprints of rhizosphere samples from KWS13, KWS14, KWS15 and MON88017 grown in Haplic Luvisol revealed a faint band with a slightly lower electrophoretic mobility of Band 1 (Band 2, Fig. S1). Band 2 occurred in all rhizosphere replicates of KWS15 and in some replicates of KWS13 and KWS14 in the (L+) treatments. Band 2 was identified by cloning, sequencing and blast analysis as *Enterobacter ludwigii* (100% identity, sequence accession no. JN836630-JN836633).

## Discussion

This is the first study on the effects of the WCR maize root feeding on both bacterial and fungal communities colonizing the maize rhizosphere. Microbial community analyses of Haplic Chernozem, Haplic Luvisol and Eutric Vertisol revealed that the three different soils harbored distinct bacterial and fungal communities. A significant rhizosphere effect of all maize lines was observed for both bacterial and fungal communities in each soil type. The influence of the maize rhizosphere was more pronounced on bacterial communities than on fungal communities. These results indicated either that the fungi were less affected by maize root exudates than the bacteria or that the resolution power of the ITS region is lower than the 16S rRNA gene fragment. The effect of the soil type and of the maize rhizosphere on the microbial community structure was already reported in several studies [37], [39], [40], [41]. However, maize line effects on the composition of bacterial and fungal communities in the maize rhizosphere shown in this study were not observed by others [41], [42]. DGGE fingerprinting and statistical analysis revealed that rhizosphere fungal communities were significantly affected by WCR larval feeding in all soil types and according to the maize line. Bacterial communities in the maize rhizosphere were more responsive than the fungal communities to larval feeding: Pronounced shifts of the bacterial communities were observed in the rhizosphere of all tested maize lines in all soil types with just one exception for MON88017 in Haplic Chernozem (Table 2). An extension of the permutation method described in Kropf et al. (2004) for two-factorial designs used to analyze interaction among larvae, maize line and bacterial or fungal communities revealed significant effects (Table S4).

Interestingly, DGGE profiles of the bacterial communities displayed a band with strongly increased intensity (Fig. 4) in the rhizosphere samples of all (L+) treatments in Haplic Chernozem. A band with the same electrophoretic mobility was observed in the rhizosphere of maize plants grown in the other two soils upon WCR attack, but not for all maize lines. Remarkably, in the bacterial fingerprints of the rhizosphere samples of MON88017 in Haplic Chernozem this band occurred also in the (L−) treatments. Sequencing of this band from the rhizosphere bacterial fingerprints of all investigated maize lines grown in presence of WCR larvae and of MON88017 also in absence of larvae revealed that the sequence behind this band shared 100% identity with the 16S rRNA sequence of *Acinetobacter calcoaceticus*. The complete genome of this strain described as a phenol degrader was recently published [43]. Poerschmann et al. [44] showed that roots of MON88017 have a higher total lignin content compared to the isogenic line. Lignin is a phenolic compound and the secretion of phenolic compounds such as t-cinnamic acid by barley plant roots was recently introduced as a novel belowground plant defense mechanism [45]. Thus, we speculate that the presence of *A. calcoaceticus* in the rhizosphere of all maize lines in presence of WCR larvae might be due to the exudation of phenolic compounds triggered by larval feeding. The presence of *A. calcoaceticus* in the rhizosphere of MON88017 even without larval feeding might be due to the higher lignin content of the root tissues which might support such phenol degrading microorganism in the rhizosphere of the transgenic line. Recently it has been reported that WCR larvae are resistant to higher levels of 2,4-dihydroxy-7-methoxy-1,4-bezoxacin-3-one (DIMBOA), a compound specifically enriched in the nutritional superior crown roots [46] and previously regarded as contributing to the resistance of some maize cultivars against larval feeding [47].

In presence of WCR larvae, a second bacterial population identified as *Enterobacter ludwigii* increased in abundance in the rhizosphere of several maize lines in Haplic Luvisol. This *Gammaproteobacterium* was originally isolated from the rhizosphere of tomato plants and was shown to display *in vitro* and *in planta* a strong antagonistic activity towards a range of fungal and oomycete pathogens [48]. Future work needs to clarify the role of *A. calcoaceticus* and *E. ludwigii* in the interaction between maize roots and WCR larvae.

Larval development, investigated by Kurtz [49] during the course of the same experiment, was influenced by the maize line in a soil type dependent manner. As expected, larval survival was drastically reduced in all three soils for MON88017. However, the microbial communities in the rhizosphere of the transgenic maize MON88017 were also influenced by the presence of WCR larvae. These changes might be mediated by plant defenses to herbivorous insects. For instance, upon WCR larval damage, roots of European maize lines (*Zea mays* L.) were reported to release the volatile compound sesquiterpene (E)-ß-caryophyllene. This compound is a strong attractant for the natural WCR enemy *Heterorhabditis megidis*, an entomopathogenic nematode [50], [51]. Plants also respond to belowground herbivore attack by the expression of the root herbivore-induced shoot resistance (RISR), resulting in a systemic response against further attacks from other groups of herbivorous insects. Recently, there has been a first report of an aboveground resistance against the nectrophic fungus *Setosphaeria turcica* [52] triggered by WCR root feeding. The findings of the present study suggest that WCR larval root feeding might cause changes in the rhizosphere microbial communities. So far, the influence of the rhizosphere community on plant-belowground herbivore interactions has been investigated only in few studies addressing the effects of soil-borne microorganisms on aboveground herbivores [53], [54]. Beneficial effects of microbial communities for plants have been shown via promoting plant growth or inducing defenses against herbivore feeding. In most cases the changes in root exudates triggered by microorganisms are regarded as a defense mechanism against soil plant pathogens [55]. In this study we found evidence that the feeding activity of WCR larvae influenced the composition of the rhizosphere microbial communities most likely by secreting phenolic compounds due to wounding. We regard the plant response to WCR feeding as the overriding factor determining the shifts in the microbial community response. Whether the changes in the bacterial and fungal communities in response to WCR feeding influence also the feeding behavior of WCR larvae or contribute to reduced damage on the roots, acting as a plant induced defense mechanism, remains to be investigated.

## Supporting Information

Figure S1 **DGGE fingerprints of 16S rRNA gene fragments PCR-amplified from TC DNA extracted from Haplic Luvisol (HL) and rhizosphere samples of four maize lines grown in HL in presence (L+) or in absence (L−) of WCR larval feeding.** M: Bacterial marker [34]. Maize lines: KWS13, KWS14, KWS15 and MON88017 (MON). Independent replicates are labeled 1 to 4. Arrows pointing to bands 1 and 2 were identified as *Acinetobacter calcoaceticus* and *Enterobacter ludwigii*, respectively.
(TIFF)

Table S1 **Geographic locations of the soil sampling areas, land use, soil texture and physico-chemical parameters.** Soil texture and the physico-chemical parameters were determined by the Institute of Soil Science (Georg-August-University, Göttingen, Germany).
(DOCX)

Table S2 **Percentage dissimilarity (** ***D*** **) and significant values (** ***P*** **) of fungal and bacterial communities fingerprints between the soil types Haplic Chernozem, Haplic Luvisol, and Eutric Vertisol.** HC: Haplic Chernozem; HL: Haplic Luvisol; EV: Eutric Vertisol. *P* values were obtained by Permutation testing with 10.000 numbers of simulations. Values of *P*<0.05 indicate significant differences between soils. Values in bold show significant differences in the microbial DGGE fingerprints between soils.
(DOCX)

Table S3 **Percentage dissimilarity (** ***D*** **) and significance values (** ***P*** **) of fungal and bacterial fingerprints in the soil and in the rhizosphere of different maize lines grown in Haplic Chernozem, Haplic Luvisol, and Eutric Vertisol, respectively.** *P* values were obtained by Permutation testing with 10.000 numbers of simulations. *P* values<0.5 indicate a significant rhizosphere effect.
(DOCX)

Table S4 **Significance values (** ***P*** **) showing the effects of the plant line, WCR larvae, and of both factors on the rhizosphere fungal or bacterial communities in the soil types Haplic Chernozem, Haplic Luvisol, and Eutric Vertisol.** HC: Haplic Chernozem; HL: Haplic Luvisol; EV: Eutric Vertisol. The multivariate statistical analysis was performed by an extension of the permutation method described in Kropf et al. [38]. Values of *P*<0.05 indicate significant differences. Bold values indicate significant differences.
(DOCX)
