# Supplementary figures and images for: Multitrophic Interaction in the Rhizosphere of Maize: Root Feeding of Western Corn Rootworm Larvae Alters the Microbial Community Composition
Source: PLoS One. 2012 May 22;7(5):e37288. doi: 10.1371/journal.pone.0037288 (PMC3358342; doi:10.1371/journal.pone.0037288)

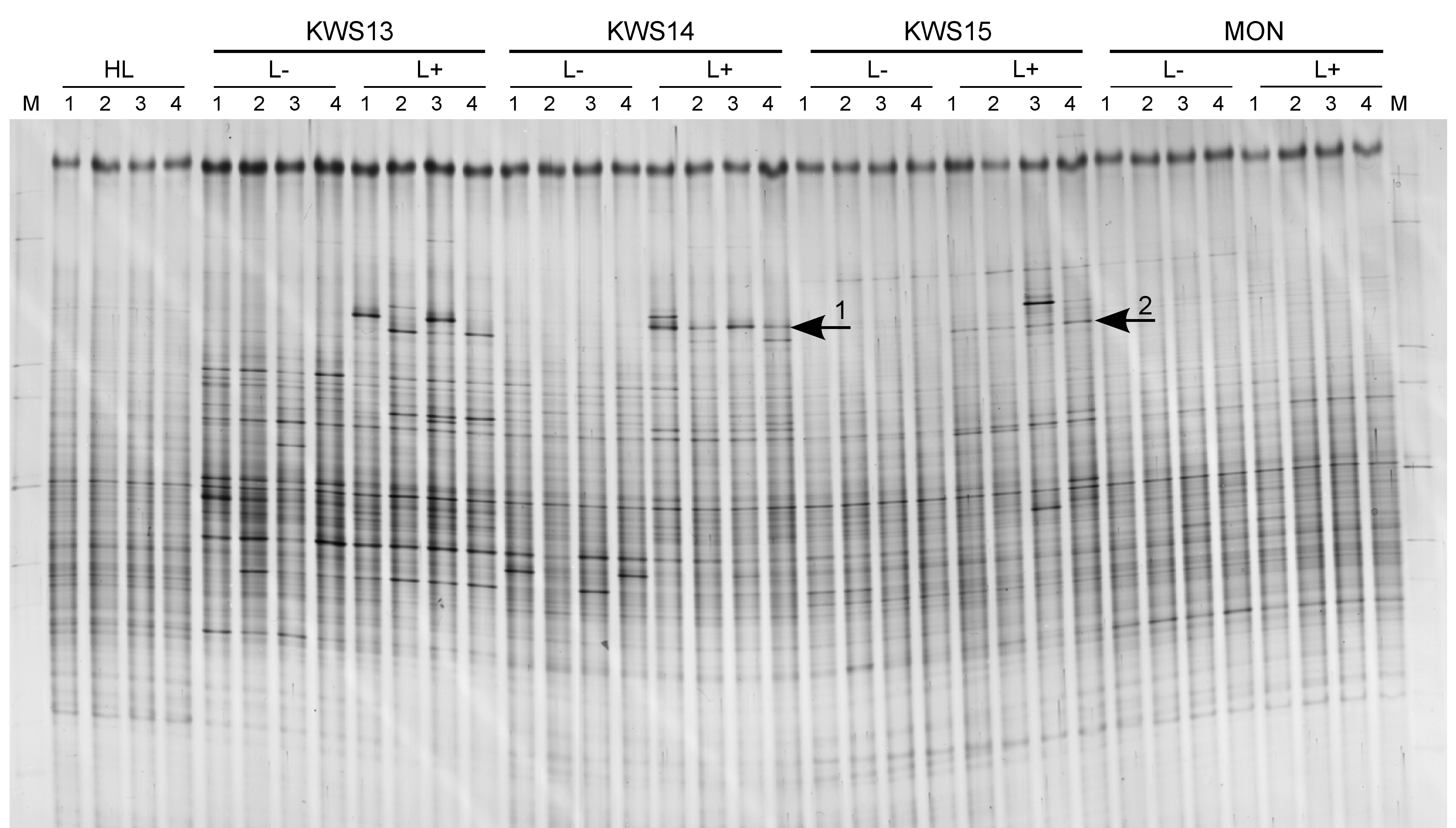

Supplement: Figure S1 — DGGE fingerprints of 16S rRNA gene fragments PCR-amplified from TC DNA extracted from Haplic Luvisol (HL) and rhizosphere samples of four maize lines grown in HL in presence (L+) or in absence (L−) of WCR larval feeding. M: Bacterial marker [34]. Maize lines: KWS13, KWS14, KWS15 and MON88017 (MON). Independent replicates are labeled 1 to 4. Arrows pointing to bands 1 and 2 were identified as Acinetobacter calcoaceticus and Enterobacter ludwigii, respectively. (TIFF) [file pone.0037288.s001.tiff]
