# Supplementary material for: Multitrophic Interaction in the Rhizosphere of Maize: Root Feeding of Western Corn Rootworm Larvae Alters the Microbial Community Composition
Source: PLoS One. 2012 May 22;7(5):e37288. doi: 10.1371/journal.pone.0037288 (PMC3358342; doi:10.1371/journal.pone.0037288)
Supplement: Table S1 — Geographic locations of the soil sampling areas, land use, soil texture and physico-chemical parameters. Soil texture and the physico-chemical parameters were determined by the Institute of Soil Science (Georg-August-University, Göttingen, Germany). (DOCX) [file pone.0037288.s002.docx]

Table S1. Geographic locations of the soil sampling areas, land use, soil texture and physico-chemical parameters

|  | **HaplicChernozem**  **(silt)** | **Haplic Luvisol**  **(silt loam)** | **Eutric Vertisol**  **(silt loam)** |
| --- | --- | --- | --- |
| **Coordinates** | 51°30`29.44 N  9°55`38.26 E | 51°29`52.88 N  9°55`38.26 E | 51°28`26.99 N  9°59`55.13 E |
| **Land use** | winter wheat | grass land | winter wheat |
| **Elevation in m** | 265 | 153 | 165 |
| **Sand content (%)** | 3. 7 | 20.7 | 11.3 |
| **Silt content (%)** | 83.8 | 68.1 | 67.8 |
| **Clay content (%)** | 12.6 | 11.2 | 20.9 |
| **pH (H_2_O)** | 7.6 | 7.6 | 7.4 |
| **C/N ratio** | 16.3 | 15.8 | 13.2 |

Soil texture and the physico-chemical parameters were determined by the Institute of Soil Science (Georg-August-University, Göttingen, Germany).
