# Supplementary material for: Multitrophic Interaction in the Rhizosphere of Maize: Root Feeding of Western Corn Rootworm Larvae Alters the Microbial Community Composition
Source: PLoS One. 2012 May 22;7(5):e37288. doi: 10.1371/journal.pone.0037288 (PMC3358342; doi:10.1371/journal.pone.0037288)
Supplement: Table S2 — Percentage dissimilarity ( D ) and significant values ( P ) of fungal and bacterial communities fingerprints between the soil types Haplic Chernozem, Haplic Luvisol, and Eutric Vertisol. HC: Haplic Chernozem; HL: Haplic Luvisol; EV: Eutric Vertisol. P values were obtained by Permutation testing with 10.000 numbers of simulations. Values of P<0.05 indicate significant differences between soils. Values in bold show significant differences in the microbial DGGE fingerprints between soils. (DOCX) [file pone.0037288.s003.docx]

Table S2. Percentage dissimilarity (*D*) and significant values (*P*) of fungal and bacterial communities fingerprints between the soil types Haplic Chernozem, Haplic Luvisol, and Eutric Vertisol.

|  | **Fungi** | | **Bacteria** | |
| --- | --- | --- | --- | --- |
| **Soil type** | *D* | *P* | *D* | *P* |
| HC/HL | 24.4 | **0.02** | 18.9 | **0.02** |
| HC/EV | 20.4 | **0.01** | 32.7 | **0.02** |
| HL/EV | 16.7 | **0.01** | 21.9 | **0.04** |

Haplic Chernozem (HC), Haplic Luvisol (HL), and Eutric Vertisol (EV). *P* values were obtained by Permutation testing with 10.000 numbers of simulations. Values of *P* <0.05 indicate significant differences between soils. Values in bold show significant differences in the microbial DGGE fingerprints between soils.
