# Supplementary material for: Multitrophic Interaction in the Rhizosphere of Maize: Root Feeding of Western Corn Rootworm Larvae Alters the Microbial Community Composition
Source: PLoS One. 2012 May 22;7(5):e37288. doi: 10.1371/journal.pone.0037288 (PMC3358342; doi:10.1371/journal.pone.0037288)
Supplement: Table S3 — Percentage dissimilarity ( D ) and significance values ( P ) of fungal and bacterial fingerprints in the soil and in the rhizosphere of different maize lines grown in Haplic Chernozem, Haplic Luvisol, and Eutric Vertisol, respectively. P values were obtained by Permutation testing with 10.000 numbers of simulations. P values<0.5 indicate a significant rhizosphere effect. (DOCX) [file pone.0037288.s004.docx]

Table S3. Percentage dissimilarity (*D*) and significance values (*P*) of fungal and bacterial fingerprints in the soil and in the rhizosphere of different maize lines grown in Haplic Chernozem, Haplic Luvisol, and Eutric Vertisol, respectively

|  | **Haplic Chernozem** | | **Haplic Luvisol** | | **Eutric Vertisol** | |
| --- | --- | --- | --- | --- | --- | --- |
|  | *D* | *P* | *D* | *P* | *D* | *P* |
| ***Fungi:Soil/Rh*** | | | | | | |
| KWS 13 | 14.2 | **0.03** | 17.2 | **0.03** | 11.6 | **0.03** |
| KWS 14 | 16.1 | **0.03** | 17.1 | **0.03** | 16 | **0.03** |
| KWS 15 | 9.8 | **0.03** | 3 | **0.03** | 8.6 | **0.03** |
| MON88017 | 14 | **0.03** | 5.5 | **0.03** | 12.5 | **0.03** |
| ***Bacteria: Soil/Rh*** | | | | | | |
| KWS 13 | 16.6 | **0.03** | 44.7 | **0.03** | 37 | **0.03** |
| KWS 14 | 10 | **0.03** | 38.3 | **0.03** | 41.5 | **0.03** |
| KWS 15 | 20.4 | **0.03** | 28 | **0.03** | 43.8 | **0.03** |
| MON88017 | 30 | **0.03** | 30 | **0.03** | 58.4 | **0.03** |

*P* values were obtained by Permutation testing with 10.000 numbers of simulations. *P* values <0.5 indicate a significant rhizosphere effect.
