# Supplementary material for: Multitrophic Interaction in the Rhizosphere of Maize: Root Feeding of Western Corn Rootworm Larvae Alters the Microbial Community Composition
Source: PLoS One. 2012 May 22;7(5):e37288. doi: 10.1371/journal.pone.0037288 (PMC3358342; doi:10.1371/journal.pone.0037288)
Supplement: Table S4 — Significance values ( P ) showing the effects of the plant line, WCR larvae, and of both factors on the rhizosphere fungal or bacterial communities in the soil types Haplic Chernozem, Haplic Luvisol, and Eutric Vertisol. HC: Haplic Chernozem; HL: Haplic Luvisol; EV: Eutric Vertisol. The multivariate statistical analysis was performed by an extension of the permutation method described in Kropf et al. [38]. Values of P<0.05 indicate significant differences. Bold values indicate significant differences. (DOCX) [file pone.0037288.s005.docx]

Table S4. Significance values (*P*) showing the effects of the plant line, WCR larvae, and of both factors on the rhizosphere fungal or bacterial communities in the soil types Haplic Chernozem, Haplic Luvisol, and Eutric Vertisol

|  | **Soil** | **Comparison** | **Plant genotype**  **(***P***)** | **WCR larvae (***P***)** | **WCR larvae x plant genotype (***P***)** |
| --- | --- | --- | --- | --- | --- |
| ***Bacteria*** | HC | HC/(KWS)s/MON | **0.0232** | **0.0001** | 0.0765 |
|  | HL | HL/(KWS)s/MON | **0.0001** | **0.0002** | **0.0012** |
|  | EV | EV/(KWS)s/MON | **0.0001** | **0.0005** | **0.0001** |
| ***Fungi*** |  |  |  |  |  |
|  | HC | HC/MON/KWS 13 | **0.0005** | **0.0030** | **0.0056** |
|  |  | HC/MON/KWS 14 | **0.0005** | **0.0007** | **0.0001** |
|  |  | HC/KWS 14/KWS 15 | **0.0002** | **0.0100** | **0.0015** |
|  | HL | HL/KWS 13/KWS 14 | **0.0001** | **0.0009** | **0.0002** |
|  |  | HL/MON/KWS 15 | **0.0123** | 0.6446 | **0.0017** |
|  | EV | EV/KWS 13/KWS 14 | **0.0005** | **0.0083** | **0.0120** |
|  |  | EV/MON/KWS 15 | **0.0003** | **0.0119** | **0.0055** |

HC: Haplic Chernozem; HL: Haplic Luvisol; EV: Eutric Vertisol. The multivariate statistical analysis was performed by an extension of the permutation method described in Kropf et al. [38]. Values of *P* < 0.05 indicate significant differences. Bold values indicate significant differences.
